# Causal links between gut microbiomes, cytokines and risk of different subtypes of epilepsy: a Mendelian randomization study

**DOI:** 10.3389/fnins.2024.1397430

**Published:** 2024-05-24

**Authors:** Youjia Qiu, Bingyi Song, Minjia Xie, Yuchen Tao, Ziqian Yin, Menghan Wang, Chao Ma, Zhouqing Chen, Zhong Wang

**Affiliations:** ^1^Department of Neurosurgery, The First Affiliated Hospital of Soochow University, Suzhou, China; ^2^Suzhou Medical College of Soochow University, Suzhou, China

**Keywords:** gut microbiome, subtype of epilepsy, cytokines, Mendelian randomization, mapped genes, FUMA

## Abstract

**Objective:**

Recent research suggests a potential link between the gut microbiome (GM) and epilepsy. We undertook a Mendelian randomization (MR) study to determine the possible causal influence of GM on epilepsy and its various subtypes, and explore whether cytokines act as mediators.

**Methods:**

We utilized Genome-Wide Association Study (GWAS) summary statistics to examine the causal relationships between GM, cytokines, and four epilepsy subtypes. Furthermore, we assessed whether cytokines mediate the relationship between GM and epilepsy. Significant GMs were further investigated using transcriptomic MR analysis with genes mapped from the FUMA GWAS. Sensitivity analyses and reverse MR were conducted for validation, and false discovery rate (FDR) correction was applied for multiple comparisons.

**Results:**

We pinpointed causal relationships between 30 GMs and various epilepsy subtypes. Notably, the Family Veillonellaceae (OR:1.03, 95%CI:1.02–1.05, *p* = 0.0003) consistently showed a strong positive association with child absence epilepsy, and this causal association endured even after FDR correction (*p*-FDR < 0.05). Seven cytokines were significantly associated with epilepsy and its subtypes. A mediating role for cytokines has not been demonstrated. Sensitivity tests validated the primary MR analysis outcomes. Additionally, no reverse causality was detected between significant GMs and epilepsy. Of the mapped genes of notable GMs, genes like BLK, FDFT1, DOK2, FAM167A, ZSCAN9, RNGTT, RBM47, DNAJC21, SUMF1, TCF20, GLO1, TMTC1, VAV2, and RNF14 exhibited a profound correlation with the risk factors of epilepsy subtypes.

**Conclusion:**

Our research validates the causal role of GMs and cytokines in various epilepsy subtypes, and there has been no evidence that cytokines play a mediating role between GM and epilepsy. This could provide fresh perspectives for the prevention and treatment of epilepsy.

## 1 Introduction

Epilepsy is a widespread and severe neurological disorder marked by sudden, simultaneous abnormal neuron activity in the brain. The rate of active epilepsy typically ranges between 4 and 12 per 1,000 (Thijs et al., 2019). Epilepsy’s incidence shows a bimodal distribution, with the highest risk seen in infants and the elderly (Thijs et al., 2019). Epilepsy remains a significant cause of disability and death, posing a considerable global societal burden. It can be categorized into focal epilepsy (FE) and generalized epilepsy (GE). The latter is subdivided into motor and non-motor (absence) epilepsy. In GE, the epileptogenic networks are widespread, covering thalamocortical structures bilaterally (Asadi-Pooya, 2019). By contrast, FE networks are limited to a single hemisphere, often involving limbic or neocortical regions (Allers et al., 2015). Both childhood absence epilepsy (CAE) and juvenile absence epilepsy (JAE) are part of an epileptic syndrome, commonly presenting with brief staring, rhythmic blinking, or motor automatisms and changes in the electroencephalogram (Kessler and McGinnis, 2019). While 65% of epilepsy patients find relief with antiepileptic medication (Kwan et al., 2011), about a third face drug resistance (Perucca et al., 2023). The frequent seizures, cognitive challenges, psychological disorders, and drug side effects profoundly impact these patients’ quality of life (Wang et al., 2023). Therefore, further exploring the etiology of epilepsy and new therapeutic options for patients has become necessary.

Recently, mounting evidence indicates a strong link between gut microbiomes (GMs) and human health (Järbrink-Sehgal and Andreasson, 2020). Research reveals that GM affects the central nervous system’s physiology, neurochemistry, behavior, and cognitive growth. The theory suggests that communication between the GM and the brain is facilitated through diverse channels, including the central and enteric nervous systems and endocrine and immune pathways (Grenham et al., 2011). This intricate relationship, the gut-brain axis, is maintained through multiple signaling pathways and connectivity networks. Studies have highlighted the role of GM and the gut-brain axis in neurological disorders such as depression, Alzheimer’s disease, and Parkinson’s disease. The hypothesis has been advised that GM dysbiosis is linked to development of epilepsy (Sorboni et al., 2022). The composition of GM in individuals with epilepsy is different from that of healthy control subjects, both before and after treatment (Gong et al., 2022). Additionally, disturbances at the level of specific GM taxa can influence the activity of neurons associated with epilepsy (Darch and McCafferty, 2022). The ketogenic diet (KD), a non-pharmacological option for those with treatment-resistant epilepsy (Socała et al., 2021), can reduce seizure attacks by 50%, as observed in 22–55% of adults and 60–75% of children (Özcan et al., 2022). Yet, whether alteration in GMs induced by KD is protective or detrimental remains debatable.

In general, neuroinflammation is a normal response that helps maintain homeostasis. However, excessive or persistent neuroinflammation can lead to cellular dysfunction (Xanthos and Sandkühler, 2014). Related studies have shown that neuroinflammatory responses are not only the result of epileptic seizures or brain neuropathology but are involved in their production (Prabowo et al., 2013; Pauletti et al., 2019). Activated microglia and astrocytes are the major cell types contributing to neuroinflammation (Vezzani et al., 2011, 2015). Several bioactive products from the GM can trigger the release of inflammatory factors by activating microglia (Kim et al., 2012). Therefore, we hypothesized that cytokines may be mediators in the pathway from GM to epilepsy. Cytokines are inflammatory regulators and, therefore, important intermediate phenotypes in inflammatory diseases. The pro-inflammatory cytokines IL-1β and IL-6 are important contributors to the inflammatory response in the brain. There is evidence that epilepsy is associated with elevated levels of pro-inflammatory cytokines (Vezzani et al., 2013). Moreover, microglia may, with the help of the above cytokines, play a role in the pathology of epilepsy through the IL-1 receptor/Toll-like receptor signaling, COX-2, and the TGF-β signaling process (Vezzani et al., 2016; Alvim et al., 2021; Soltani Khaboushan et al., 2022).

Robust evidence from randomized controlled trials (RCTs) verifies the connection between GM dysfunction and epilepsy onset. However, the nature of this relationship, whether causal or not, requires further study. Mendelian randomization (MR) is an advanced statistical method designed to explore causative associations between variables. It uses single nucleotide polymorphisms (SNPs) as instrumental variables. This method can help negate reverse causality and confounding factor effects. Still, no MR studies have focused on children with juvenile absence epilepsy, who often exhibit higher drug resistance (Symonds et al., 2021). Thus, our research employed a MR analysis using data from a published Genome-Wide Association Study (GWAS) to explore the potential causal link between GM and various epilepsy subtypes.

## 2 Materials and methods

### 2.1 Ethical approval

The GWAS data utilized in this study were publicly available and lacked identifiable details. The data had already been approved by an ethics committee, eliminating the need for additional ethical clearance for our research.

### 2.2 Study design

**Figure 1:**
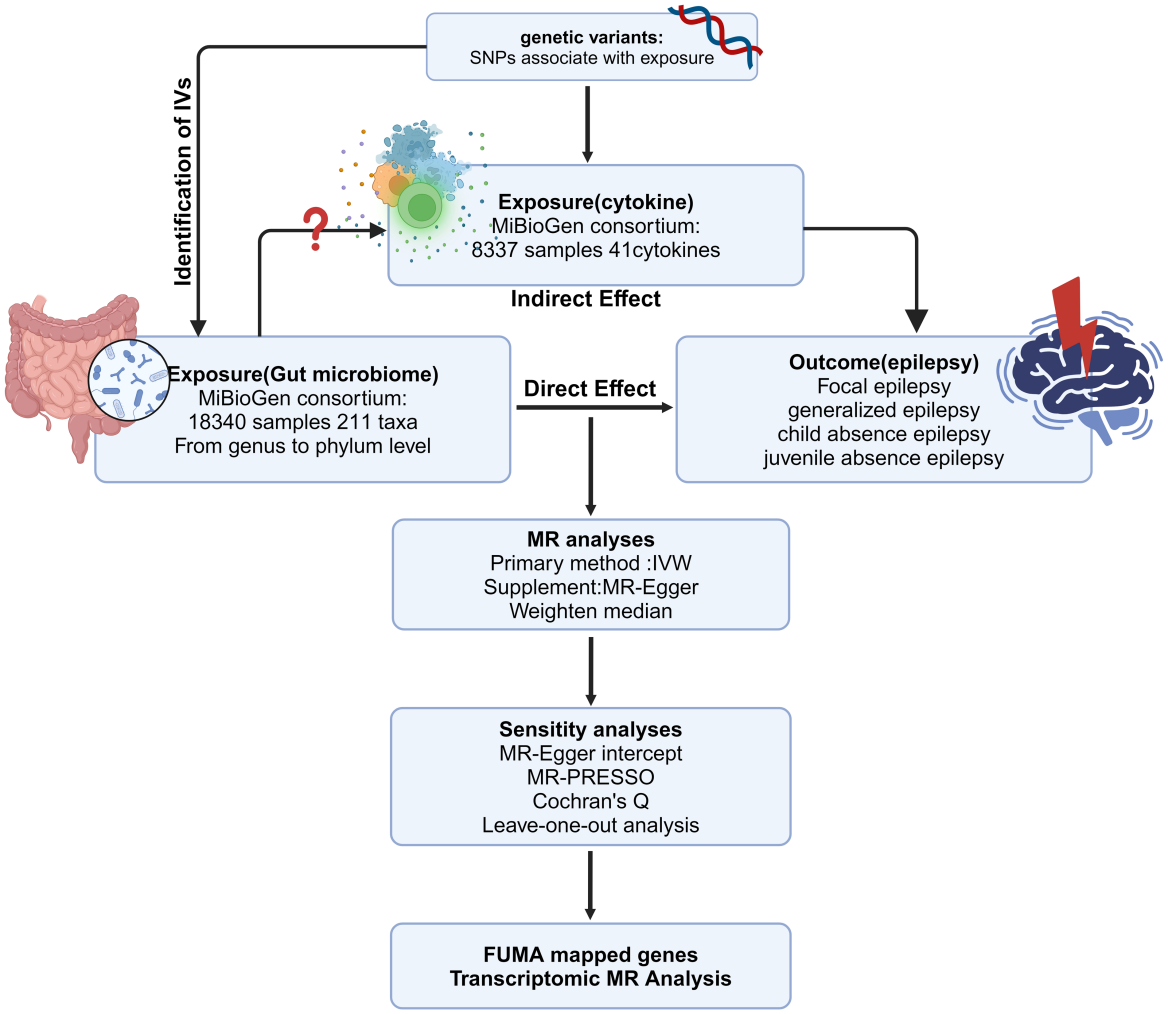
The overview of the MR analysis design (Created with BioRender.com). IV, instrumental variable; MR, Mendelian randomization; SNPs, single-nucleotide polymorphisms; IVW, inverse-variance weighted; MR-PRESSO, MR pleiotropy residual sum and outlier.

We employed two-sample MR to explore the relationship between GM and various epilepsy subtypes such as FE, GE, JAE, and CAE. Our research follows STROBE-MR guidance (Supplementary Table S1). The study’s flowchart is depicted in Figure 1. For reliable MR study outcomes, three criteria must be met: (1) There must be a significant association between genetic variants and the specific exposure. (2) Genetic variants must not be linked to confounding factors associated with the outcomes. (3) Genetic variants should only affect the outcomes through exposure factors and no alternative pathways. IVs satisfying these criteria are deemed fit for MR analysis, ensuring accurate causal inference.

### 2.3 Data source

Summary statistics for GMs were sourced from an extensive GWAS by the MiBioGen consortium, which included 18,340 individuals of European ethnic backgrounds across 11 countries and analyzed 122,110 genetic variation loci (Kurilshikov et al., 2021). We accessed data on 211 gut microbial taxa, covering five taxonomic levels from genus to phylum. The adjustment was made for sex, age, study-specific covariates, and the primary genetic principal components reflecting population stratification (Kurilshikov et al., 2021). GWAS data for FE and GE were procured from FinnGen Research (https://r9.finngen.fi/) and released in September 2022. Epilepsy diagnoses in the FinnGen dataset used the G40 code from the 10th edition of the International Classification of Diseases (ICD). The summary data for CAE and JAE came from the IEU open GAWS project (https://gwas.mrcieu.ac.uk/). As data on GMs and different subtypes of epilepsy were obtained from different databases, the populations with GMs and epilepsy did not overlap. Genetic data for cytokines were from a previous GWAS (8,337 individuals) and included 41 inflammatory cytokines (Ahola-Olli et al., 2017). This study amalgamated findings from both The Cardiovascular Risk in Young Finns Study and FINRISK surveys, wherein participants averaged 37 years and 60 years, respectively. Under the framework of our MR design, a comprehensive summary of the exposures and outcomes subjected to analysis is provided in Table 1.

**Table 1:** Details of the exposure and outcome.

**Trait**	**Consortium**	**Samples**	**Case**	**Control**
**Exposure**				
211 GM taxa	MiBioGen	18,340	/	/
41 cytokines	Ahola-Olli et al.	8,337	/	/
**Outcome**				
Generalized epilepsy	FinnGen (R9)		2,632	300,631
Focal epilepsy	FinnGen (R9)		1,297	300,631
Child absence epilepsy	IEU OpenGWAS project	30,470	793	29,677
Juvenile absence epilepsy	IEU OpenGWAS project	30,092	415	29,677

GM, gut microbiome; GWAS, genome-wide association study.

### 2.4 Identification of IVs

To enhance the precision of our results, we conducted a thorough data screening process for the information extracted from MiBioGen. Owing to the limited number of IVs meeting a stringent threshold of *p* < 5 × 10^−8^, we adjusted the threshold to *p* < 1 × 10^−5^ to obtain a more substantial number of IVs derived from GMs for our analysis. By increasing the number of IVs included, the efficacy of the statistical analyses can be improved. Similarly, we set the threshold to *p* < 5 × 10^−6^ to expand the number of available IVs for each cytokine. Additionally, we set *r*^2^ to 0.01 and the clumping window width kb = 10,000 to ensure no linkage disequilibrium among IVs. Further refinements to our IVs included removing palindromic SNPs and any not present in the results, allowing us to assess whether the selected IVs had any associations with potential confounding risk factors. Lastly, the F-statistics were calculated using the subsequent formula to evaluate the potential bias arising from weak instruments: (*F* = beta^2^/se^2^).

### 2.5 Primary analyses

We used the inverse variance weighted (IVW) method as our primary means to evaluate the causal relationship. Other methods, including MR-Egger, weighted mode, weighted median, and simple mode, were also employed. MR-Egger detects and adjusts for possible horizontal pleiotropy, but its results can be skewed by the presence of outlying genetic variables (Sun et al., 2023). The weighted median method ensures causality estimate stability by correcting potential errors when up to 50% of the IVs might be invalid and may provide better causality detection than the MR-Egger under certain conditions (Morris et al., 2022). In addition, reverse MR was conducted to rule out reverse causality. The odds ratio (OR) with its 95% confidence interval (95% CI) indicates GM’s causal effect on epilepsy. We adjusted the *p*-value using the false discovery rate (FDR) procedure for multiple comparisons (Xiang et al., 2021).

### 2.6 Mediation analysis

Mediation analysis aims to evaluate the pathway from exposure to outcome through a mediator, which helps explore the potential mechanisms by which exposure affects outcome (Carter et al., 2021). After assessing causal effects using two-sample analysis, the selected GM and cytokines with significant causal impact on epilepsy will be included in the mediation analysis. If GM has a causal effect on cytokines, we will explore whether cytokines are mediators in the pathway from GM to epilepsy. Specifically, a two-step MR analysis was conducted to examine the mediating pathways from GM to epilepsy. In the first step, the IV of GM was used to estimate the causal effect of exposure on potential mediating variables. In the second step, the causal effect of mediating variables on epilepsy risk was estimated. Finally, the indirect effect of GM on epilepsy was investigated by cytokines. The two-step MR is similar to the product of coefficient methods. Two MR estimates were calculated: β1: causal effect of exposure on the mediator; β2: causal effect of the mediator on the outcome; β3: causal effect of exposure on the outcome (Relton and Davey Smith, 2012; Burgess et al., 2015). These two estimates can be multiplied to estimate indirect effects, and the percentage of mediation was calculated by applying the following formula: (β1 × β2)/(β3).

### 2.7 Sensitivity analyses

Sensitivity analyses were performed to evaluate the robustness of MR estimates. We assessed the heterogeneity of the IVs through the calculation of I^2^ statistics and the application of Cochran’s Q test. The MR-Egger intercept and MR pleiotropy residual sum and outlier (MR-PRESSO) tests were employed to pinpoint pleiotropy and to identify potential outliers with heterogeneity among the IVs (Li et al., 2023). Furthermore, a leave-one-out analysis determined if a single SNP influenced the MR findings. This approach was carried out to assess whether the estimates were affected by biases or driven by outliers (Li et al., 2023). All MR calculations were conducted using R software version 4.3.0 and the R package MendelR (7.8.0).

### 2.8 Mapping SNPs to genes

To better understand GMs’ effect on epilepsy, we integrated significant SNPs from each GM as primary SNPs in the FUMA GWAS (Watanabe et al., 2017). These SNPs were then related to genes using FUMA integrated SNPGENE tool. A protein–protein interaction (PPI) network was created using the STRING database, with a recommended minimum interaction score of 0.4 and default settings for other parameters (Szklarczyk et al., 2021). The PPI network was visualized in Cytoscape (V3.9.1).

### 2.9 Transcriptomic MR analysis

To delve deeper into the role of genes derived from the positive GMs and CAE GWAS, we executed a transcriptomic MR analysis. Cis-expression quantitative trait loci (cis-eQTL) data related to these genes were sourced from the eQTLGen Consortium (https://eqtlgen.org/), comprising 16,987 genes from an extensive pool of 31,684 blood samples, mainly from a healthy European population (Võsa et al., 2021). For cis-eQTL MR, using a very stringent correlation criterion could exclude causal variants. Hence, these expression quantitative trait loci (eQTLs) underwent clumping based on pairwise linkage disequilibrium (LD) threshold, with an r-squared (*r*^2^) value threshold of less than 0.1. Subsequently, the SNPs linked to these genes were used as IVs for MR between these IVs and epilepsy. We used FDR correction for multiple comparisons, and a result of *p*-FDR < 0.05 indicated statistical significance.

## 3 Results

### 3.1 Details of GMs

**Figure 2:**
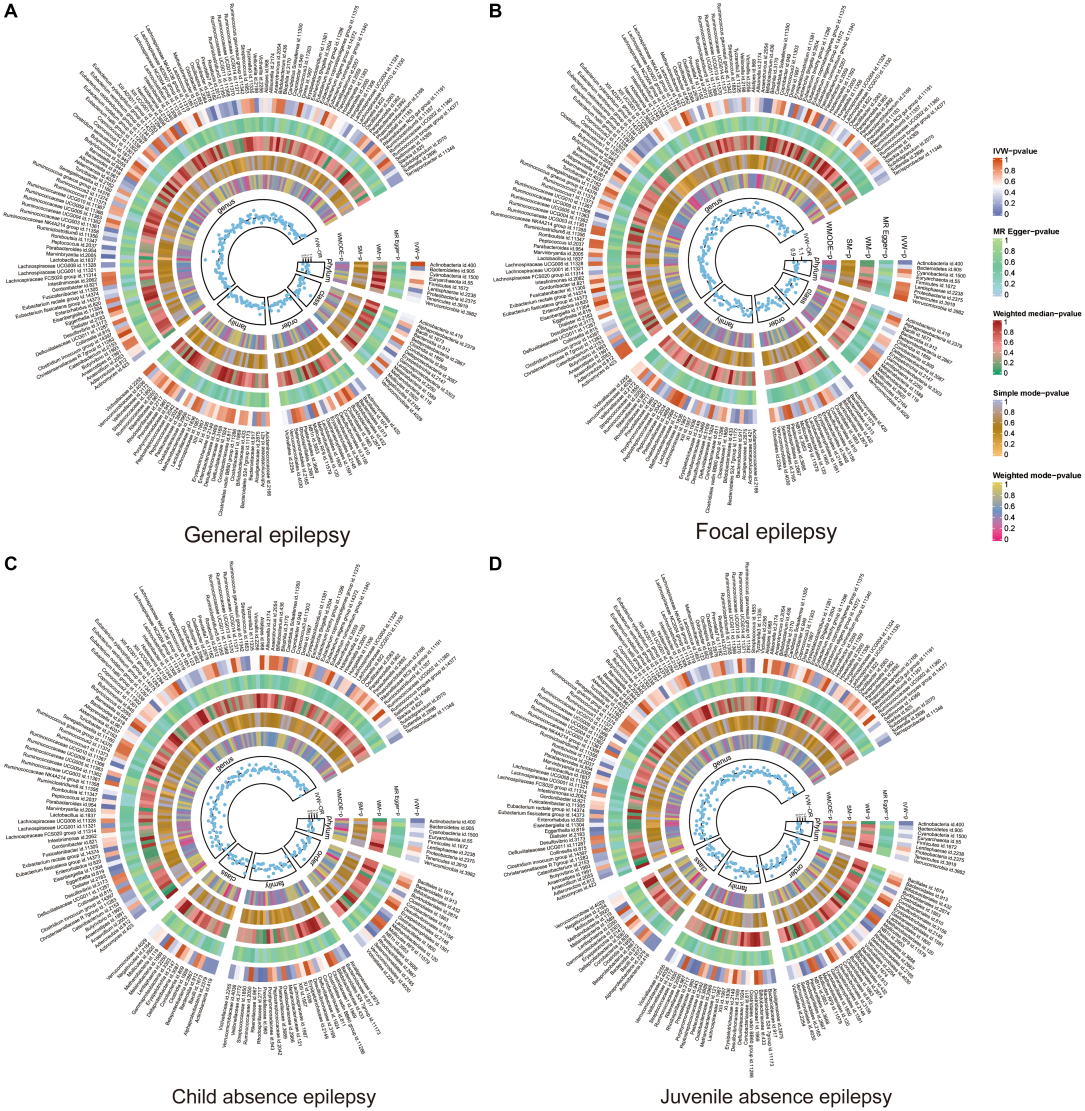
Heat map of GM taxa causally associated with subtypes of epilepsy. Red represents risk factors, while blue represents protective factors. **(A)** Heat map of GM taxa causally associated with GE; **(B)** Heat map of GM taxa causally associated with FE; **(C)** Heat map of GM taxa causally associated with CAE; **(D)** Heat map of GM taxa causally associated with JAE. GM, gut microbiome; IVW, inverse-variance weighted method; WM, weighted median; OR, odds ratios; SM, simple mode; WMODE, weighted mode.

A total of 211 GMs spanning five biological levels were included in this study. From these, 15 bacterial traits with unspecified characteristics were excluded. Consequently, 196 bacterial traits from the IEU and FinnGen datasets were incorporated into the MR analysis. The *F* value for all IVs exceeded 10, signifying no bias from weak IVs. Details about IVs can be found in Supplementary Tables S2, S3, and the MR results for the 196 GMs are presented in Figure 2 and Supplementary Table S4. Supplementary Table S5 shows the whole results of MR analysis between cytokines and epilepsy.

### 3.2 MR estimates

In FE, the genera *Bilophila* (OR: 1.24, 95% CI: 1.04–1.48, *p* = 0.02), *Bifidobacterium* (OR: 1.26, 95% CI: 1.04–1.51, *p* = 0.02), *Eubacterium nodatum group* (OR: 1.10, 95% CI: 1.01–1.21, *p* = 0.04), and *Erysipelatoclostridium* (OR:1.14, 95% CI:1.002–1.30, *p* < 0.05) were positively associated with FE, while *Flavonifractor* (OR:0.78, 95% CI:0.62–0.99 *p* = 0.04) was negatively correlated with FE. In GE, six GMs were identified. Of these, class *Gammaproteobacteria* (OR: 2.05, 95% CI: 1.02–4.14, *p* < 0.05), genus *Marvinbryantia* (OR: 2.04, 95% CI: 1.35–3.10, *p* = 0.001), genus *Oxalobacter* (OR: 1.30, 95% CI: 1.04–1.64, *p* = 0.02), genus *Ruminococcaceae Ucg013* (OR: 1.59, 95% CI: 1.02–2.45, *p* = 0.04), and order *Mollicutes RF9* (OR: 1.48, 95% CI:1.03–2.13, *p* = 0.03) were associated with an increased risk of GE; however, the genus *Phascolarctobacterium* (OR: 0.58, 95% CI: 0.37–0.91, *p* = 0.02) was associated with a risk reduction of GE.

Nine GMs were identified in CAE. The family *Veillonellaceae* (OR: 1.03, 95% CI: 1.02–1.05, *p* < 0.001), genus *Desulfovibrio* (OR: 1.03, 95% CI: 1.00–1.06, *p* = 0.03), genus *Oscillibacter* (OR: 1.02, 95% CI: 1.001–1.04, *p* = 0.04), genus *Anaerostipes* (OR: 1.03, 95% CI:1.0004–1.06, *p* < 0.05), and phylum *Verrucomicrobia* (OR:1.02, 95% CI: 1.003–1.039, *p* = 0.03) were associated with an increased risk of CAE, while the class *Bacteroidia* (OR:0.97, 95% CI:0.95–0.995, *p* = 0.02), genus *Ruminococcaceae NK4A214 group* (OR: 0.97, 95% CI:0.94–0.996, *p* = 0.03), phylum *Bacteroidetes* (OR: 0.97, 95% CI: 0.95–0.99, *p =* 0.01), and order *Bacteroidales* (OR: 0.97, 95% CI: 0.95–0.995, *p* = 0.02) were associated with a reduced risk of CAE. For JAE, 10 GMs were identified to have a causal association. Of these, the genus *Ruminococcaceae UCG004* (OR: 1.02, 95% CI: 1.003–1.03, *p* = 0.02), genus *Candidatus soleaferrea* (OR: 1.01, 95% CI: 1.001–1.02, *p* = 0.03), and genus *Lachnospiraceae UCG010* (OR: 1.02, 95% CI: 1.001–1.03, *p =* 0.04) were positively correlated with JAE; while family *Rhodospirillaceae* (OR: 0.99, 95% CI: 0.98–0.996, *p* = 0.007), family *Prevotellaceae* (OR: 0.99, 95% CI: 0.97–0.999, *p =* 0.03), genus *Parabacteroides* (OR: 0.97, 95% CI: 0.95–0.99, *p* = 0.01), genus *Ruminococcaceae UCG010* (OR: 0.98, 95% CI: 0.95–0.998, *p* = 0.03), genus *Eggerthella* (OR: 0.99, 95% CI: 0.97–0.999, *p* = 0.03), genus *Eubacterium nodatum group* (OR:0.99, 95% CI: 0.99–0.9998, *p =* 0.04), and order *Rhodospirillales* (OR: 0.99, 95% CI: 0.98–0.998, *p* = 0.02) were negatively associated with JAE. Of 30 GMs, only family *Veillonellaceae* in CAE passed FDR correction (*p*-FDR < 0.05). The MR results of positive GMs in different epilepsy subtypes are shown in Supplementary Table S6.

**Figure 3:**
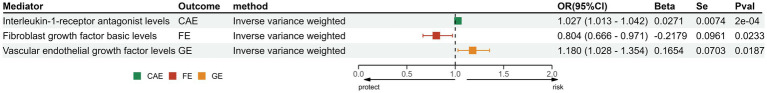
The positive results of MR analysis between cytokines and different subtypes of epilepsy. IL-1Ra, Interleukin-1-receptor antagonist levels; FGF-b, Fibroblast growth factor basic levels; VEGF, Vascular endothelial growth factor levels; SNP, single-nucleotide polymorphisms; OR, odds ratios; CI, confidence interval.

In exploring the causal relationship between cytokines and epilepsy, we found that Interleukin-1-receptor antagonist (OR: 1.03, 95% CI: 1.01–1.04, *p* < 0.001) and Vascular endothelial growth factor (OR: 1.18, 95% CI: 1.03–1.35, *p* = 0.02) have a protective effect on epilepsy. Besides, Fibroblast growth factor basic (OR: 0.80, 95% CI:0.67–0.97, *p* = 0.02) can increase the risk of FE (Figure 3).

### 3.3 Mediation analysis

In this study, both GM and cytokines were causally associated with epilepsy. This seems to indicate that cytokines play a mediating role in the pathway between GM and dementia. This mediation analysis was based on the significant correlation between GM and cytokines. However, we did not observe a causal relationship between GM and cytokines significantly associated with epilepsy (Supplementary Table S7). Therefore, there is insufficient evidence to prove that GM has an indirect effect on epilepsy through cytokines.

### 3.4 Sensitivity analyses

The MR-PRESSO and MR-Egger intercept analysis confirmed the absence of potential horizontal pleiotropy, as detailed in Table 2 and Supplementary Table S8. Based on the Cochrane Q test and *I^2^* statistics, no evidence of heterogeneity was found among the selected IVs and their relationship with epilepsy. Results from the scatter plot are provided in the Supplementary Figure S1. The leave-one-out analysis did not identify any outlier SNPs (Supplementary Figure S2). In conclusion, these findings underline a consistent and robust causal link between GM and epilepsy.

**Table 2:** Horizontal pleiotropy analysis for IVs of 30 GM taxa associated with epilepsy.

**Exposure**	**Outcome**	**MR-Egger intercept test**		**MR-PRESSO global test**	
		**Egger_ intercept**	***p*-value**	**RSS obs**	***p*-value**
Genus Phascolarctobacterium id.2168	GE	5.68	0.68	7.76	0.50
Genus Oxalobacter id.2978	GE	8.16	0.52	10.06	0.57
Genus Ruminococcaceae UCG013 id.11370	GE	10.55	0.31	11.04	0.54
Genus Marvinbryantia id.2005	GE	5.76	0.33	12.50	0.43
Order Mollicutes RF9 id.11579	GE	12.72	0.24	15.28	0.34
Class Gammaproteobacteria id.3303	GE	5.61	0.23	10.28	0.26
Genus *Eubacterium nodatum* group id.11297	FE	0.03	0.41	6.44	0.88
Genus Bifidobacterium id.436	FE	−0.01	0.47	19.22	0.17
Genus Bilophila id.3170	FE	0.02	0.60	15.34	0.40
Genus Erysipelatoclostridium id.11381	FE	−0.01	0.77	10.49	0.83
Genus Flavonifractor id.2059	FE	0.02	0.04	3.71	0.69
Family Veillonellaceae id.2172	CAE	0.00	0.69	5.29	0.94
Phylum Bacteroidetes id.905	CAE	0.00	0.67	6.41	0.70
Phylum Verrucomicrobia id.3982	CAE	0.00	0.48	4.09	0.83
Class Bacteroidia id.912	CAE	0.00	0.30	5.84	0.75
Order Bacteroidales id.913	CAE	0.00	0.30	5.84	0.76
Genus Anaerostipes id.1991	CAE	−0.01	0.37	4.94	0.48
Genus Desulfovibrio id.3173	CAE	0.04	0.40	NA	NA
Genus Ruminococcaceae NK4A214 group id.11358	CAE	0.00	0.96	1.11	0.90
Genus Oscillibacter id.2063	CAE	0.00	0.93	12.74	0.19
Family Prevotellaceae id.960	JAE	−0.01	0.09	7.96	0.71
Family Rhodospirillaceae id.2717	JAE	0.00	0.55	12.10	0.55
Order Rhodospirillales id.2667	JAE	0.00	0.72	12.79	0.48
Genus Eggerthella id.819	JAE	0.00	0.44	1.81	0.83
Genus Parabacteroides id.954	JAE	0.00	0.79	1.92	0.81
Genus Ruminococcaceae UCG004 id.11362	JAE	0.00	0.67	5.43	0.63
Genus Ruminococcaceae UCG010 id.11367	JAE	−0.01	0.42	NA	NA
Genus *Eubacterium nodatum* group id.11297	JAE	0.00	0.29	5.86	0.74
Genus Candidatus Soleaferrea id.11350	JAE	0.01	0.47	5.89	0.57
Genus Lachnospiraceae UCG010 id.11330	JAE	0.00	0.62	3.51	0.73

GE, generalized epilepsy; FE, focal epilepsy; CAE, child absence epilepsy; JAE, juvenile absence epilepsy; MR-PRESSO, MR pleiotropy residual sum and outlier.

### 3.5 Reverse Mendelian randomization

1To examine reverse causality, we considered epilepsy subtypes as exposure and significant GMs as outcomes. After adjusting for linkage disequilibrium, we sourced 17, 11, 14, and 8 SNPs associated with CAE, JAE, FE, and GE from the GWAS database. As demonstrated in Supplementary Table S9, no reverse causality between epilepsy subtypes and GMs was evident (*p* > 0.05).

### 3.6 Mapping SNPs to genes

To delve deeper into biologically significant findings, the IVs, used as genetic variants, were functionally annotated using the FUMA GWAS tool. The identified genes are listed in Supplementary Table S10. Using STRING, we constructed a PPI network for these mapped genes across different epilepsy subtypes, with outcomes displayed in Supplementary Figure S3.

### 3.7 Transcriptomic MR analysis

**Figure 4:**
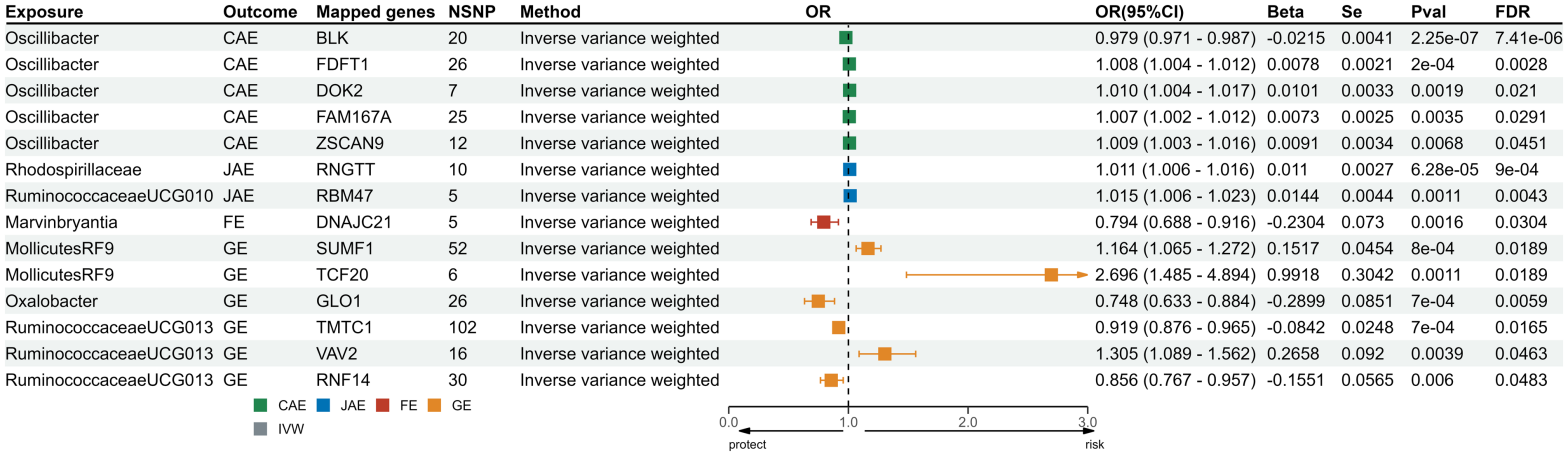
Forest plots for transcriptomic MR analysis of significant mapped genes. CAE, child absence epilepsy; JAE, juvenile absence epilepsy; FE, focal epilepsy; GE, generalized epilepsy; IVW, inverse-variance weighted method; OR, odds ratios; CI, confidence interval; FDR: false discovery rate correction.

We sourced SNPs linked to gene expression (eQTLs) from the eQTLGen consortium. Transcriptomic MR analysis of the family *Veillonellaceae* and CAE remained significant post-FDR correction. Of the 631 mapped genes, 501 were derived from cis-eQTL summary statistics. Post FDR correction, genes like FDFT1, DOK2, FAM167A, and ZSCAN9 in the genus *Oscillibacter* showed a positive correlation with CAE risk, while BLK indicated a negative correlation. For JAE, RNGTT from the family *Rhodospirillaceae* and RBM47 from the genus of *Ruminococcaceae UCG010* showed positive associations. DNAJC in genus *Erysipelatoclostridium* was associated with FE risk. In the case of GE, genes such as SUMF1 and TCF20 from order *Mollicutes RF9* and VAV2 from the genus *Ruminococcaceae UCG13* were positively associated; conversely, GLO1 from the genus *Oxalobacter* and TMTC from the genus *Ruminococcaceae UCG13* indicated a reduced risk for GE. Comprehensive results from the five methods are consolidated in Supplementary Table S11; the directionality across these methods was consistent (Figure 4). Heterogeneity testing showed nonsignificant differences, suggesting no notable heterogeneity in the MR estimates (Supplementary Table S12).

## 4 Discussion

In this study, we conducted MR analyses to investigate the potential causal relationship between GMs and different epilepsy subtypes. We found that 30 GMs were associated with four epilepsy subtypes using extensive GWAS statistics, and no reverse causality was identified. Notably, the family *Veillonellaceae* in CAE displayed a significant causal association after FDR correction. Three types of cytokines have significant effects on epilepsy. These results could provide insights into the therapeutic role of epilepsy.

The role of GM in maintaining the state of epilepsy has gained increasing recognition. Yet, due to limited clinical trials examining the differences in the metagenome/metabolome between epilepsy patients and healthy individuals, the influence of GM on epilepsy remains elusive. In our conclusions, no reverse causality was found for epilepsy on GM. Considering modifications related to several external factors (e.g., diet and age), it is not easy to conduct relevant animal or clinical studies. Although some studies have found increased alpha diversity and abundance of thick-walled phyla in patients with drug-resistant epilepsy and altered abundance of *Prevotella*, *Ruminococcus*, and other flora in patients with cerebral palsy with epilepsy (Peng et al., 2018; Huang et al., 2019). The results of the available studies are not uniform, and it is therefore difficult to define relevant criteria (Russo, 2022). Previous research has highlighted the importance of *Veillonellaceae* in developing the intestinal microecosystem during early life (Lou et al., 2022). While direct evidence connecting *Veillonellaceae* and epilepsy is scarce, its influence on normal brain functions is documented. Furthermore, *Veillonellaceae*, part of the phylum *Firmicutes*, shows increased abundance in patients with drug-resistant epilepsy, which also decreases after antiepileptic treatment (Cheraghmakani et al., 2021). Research has identified a prevalent genus, *Marvinbryantia*, in epileptic animals, which correlates positively with excitatory neurotransmitters (Cheraghmakani et al., 2021). Our findings show that a positive causal relationship between the genus *Marvinbryantia* and GE aligns with these experimental results. Oliveira et al. observed a negative correlation between *Marvinbryantia* and d-glucose and lactate. This negative association may disrupt carbohydrate metabolism, possibly contributing to the mechanisms underlying epilepsy (Fei et al., 2020). Additionally, *Marvinbryantia* is a pro-inflammatory bacterium, possibly exacerbating inflammation and precipitating epileptic conditions (Oliveira et al., 2022). Our MR analysis highlighted *Parabacteroides*, belonging to the family *Tannerellaceae*, as a potential protective agent for JAE. Previously linked to increased ketosis and metabolic enhancement in humans (David et al., 2014), this bacterium might explain the seizure control seen with the KD (Bough and Rho, 2007). Olson et al. posited that the KD’s anti-seizure effects arise from increasing specific bacterial species, particularly *Parabacteroides merdae*. Together, these bacteria reduce the γ-glutamylation of amino acids and boost the hippocampus’s gamma-aminobutyric acid/glutamate ratio, preventing epilepsy (Olson et al., 2018).

Interestingly, 28 other GMs with nominal causal associations, such as Ruminococcus, echoed past findings. Similar to *Veillonellaceae*, *Ruminococcus*, another microbiome from phylum *Firmicutes*, increases in individuals with autism spectrum disorders (Wang et al., 2013). *Ruminococcus* causes reduced levels of N-acetyl aspartate, a marker of neuronal health, and is typically diminished in individuals diagnosed with epilepsy (Zhang et al., 2016; Mudd et al., 2017). The presence of this microorganism also correlates positively with glutamate and glutamine, both closely associated with the pathogenesis of epilepsy (Sun et al., 2016). Furthermore, *Ruminococcus* is associated with reduced levels of 5-hydroxytryptophan, which inhibits T-type calcium channels and subsequently reduces epileptiform discharges (Petersen et al., 2017). Additionally, the abundance of the phylum *Verrucomicrobia*, *Bacteroides*, and its related genus (including *Bacteroides* and *Barnesiella*) was observed to be higher in the drug-resistant epilepsy group than in the control group, aligning with our findings (Peng et al., 2018). Notably, subjects with cerebral palsy and epilepsy, potential risk factors for FE and GE, exhibited an elevated presence of *Eubacterium* (Peng et al., 2023). Moreover, *Anaerostipes* was found to be more prevalent in epilepsy patients (Gong et al., 2021). However, it is noteworthy that while *Lachnospiraceae* is more abundant in healthy individuals (Ding et al., 2021), a reduced presence was noted in temporal lobe epilepsy patients (Wei et al., 2023). This observation contrasts with our MR findings and necessitates further exploration.

Currently, the exact mechanism by which the GM contributes to epilepsy has not been determined. Although the available data do not confirm that cytokines are mediators between GM and epilepsy, we observed many results that are consistent with previous studies. As a protective factor in epilepsy, the IL-1 receptor antagonist anakinra can be used as a treatment for refractory epilepsy (Yamanaka et al., 2021), and vascular endothelial growth factor is considered an attractive target for epilepsy treatment (Lange et al., 2016). On the other hand, we have found that IL-2 increases the risk of epilepsy. As a factor that plays an important immune role, elevated levels of IL-2 over-activate signaling pathways, leading to a worse prognosis in temporal lobe epilepsy (Mazumder et al., 2019).

The precise mechanisms driving the relationship between GMs and epilepsy are not yet fully understood, but emerging evidence provides some initial insights into possible mechanisms. Various bioactive products from GMs have the potential to influence the brain either directly or indirectly. As mentioned above, GM can mediate neuroinflammation, and pro-inflammatory cytokines may contribute to epilepsy by triggering oxidative stress, increased PIC secretion and BBB disruption (Vezzani et al., 2013). For instance, lipopolysaccharides can directly affect the central nervous system by activating Toll-like receptors on microglia and triggering the release of inflammatory factors (Kim et al., 2012). In addition, enteroendocrine signaling and microbial metabolism are among the mechanisms that may contribute to epilepsy. By increasing the release of excitatory neurotransmitters such as glutamate and decreasing inhibitory neurotransmitters such as GABA, neurotransmission can be disrupted (Su et al., 2015; Rana and Musto, 2018). SCFAs are also thought to be protective against epilepsy (Ding et al., 2021). From an autoimmune perspective, GM can regulate microglia maturation as well as astrocyte activation (de Theije et al., 2011). This implies that GM can regulate epilepsy development by modulating innate immunity, adaptive immunity and inflammatory mechanisms (Ding et al., 2021). Notably, *Bacteroidetes* have been linked to conditions such as encephalitis and autoimmunity (Xu et al., 2020), which could play a role in epilepsy development. A theory also suggests that GMs might affect the hypothalamic–pituitary–adrenal axis, thereby increasing the likelihood of epilepsy (Sudo et al., 2004).

Combined with existing theories, our study has profound clinical implications and provides new ideas for the clinical management of epilepsy. The risk of epilepsy can be reduced by the use of probiotics. In the field of pharmacological microbiology, GMs and their secreted cytokines can be used as drug targets. He et al. used FMT to improve epilepsy in a 22-year-old girl (He et al., 2017). Additionally, GMs may serve as promising biomarkers for identifying the prognosis of epilepsy patients. For instance, a smaller Bacteroidetes/Firmicutes ratio may increase the risk of seizures and lead to poor prognosis (Citraro et al., 2021). The different abundance composition of GMs in the intestine can also be an indicator for evaluating the efficacy of KD (Xie et al., 2017). Furthermore, this study also has several strengths. First, it comprehensively examines the causal relationship between GM and various epilepsy subtypes. Unlike observational studies, this analysis is less vulnerable to confounding factors and reverse causality. Second, the paper’s statistical robustness is evident with a substantial GWAS data sample size for exposure and outcome and significant estimated effects for each genetic variable (*F*-value >10). Besides, a rigorous FDR correction was applied in the MR analysis to minimize type I errors. Third, we included cytokines to analyze possible mediating factors from GM to epilepsy and annotated genetic variants through FUMA. However, certain limitations warrant consideration. The GWAS data on GMs encompass various diseases and age groups without specific gender and age stratification. We included only individuals of European descent due to the absence of large-scale GWAS data for other ancestries, which might make these findings less generalizable. Lastly, while the MR analysis parallels the insights from RCT studies, further validation through animal experiments remains essential.

## 5 Conclusion

In conclusion, our MR study underscores potential causal connections between GMs and various epilepsy subtypes (GE, FE, JAE, and CAE), suggesting that the dysbiosis of the family *Veillonellaceae* might play a role in CAE onset. A mediating role of the cytokines has not been shown to exist between GM and epilepsy. Further studies are essential to understand the potential mechanisms by which GM might influence epilepsy treatment.

## Data availability statement

The original contributions presented in the study are included in the article/Supplementary material, further inquiries can be directed to the corresponding authors.

## Author contributions

YQ: Conceptualization, Formal analysis, Writing – original draft. BS: Conceptualization, Formal analysis, Writing – original draft. MX: Data curation, Writing – original draft. YT: Data curation, Writing – original draft. ZY: Methodology, Software, Writing – original draft. MW: Methodology, Software, Writing – original draft. CM: Supervision, Validation, Writing – review & editing. ZC: Supervision, Validation, Writing – review & editing. ZW: Funding acquisition, Resources, Supervision, Writing – review & editing.
