# Supplementary material for: Causal links between gut microbiomes, cytokines and risk of different subtypes of epilepsy: a Mendelian randomization study
Source: Front Neurosci. 2024 May 24;18:1397430. doi: 10.3389/fnins.2024.1397430 (PMC11157073; doi:10.3389/fnins.2024.1397430)
Supplement: Supplementary file 1 [file Data_Sheet_1.ZIP › Supplementary Materials/Supplementary Figures.pdf]

Causal Links Between Gut Microbiomes and Risk of Different Subtypes of Epilepsy: A Mendelian Randomization Study

Youjia Qiu[1, #], Bingyi Song[1, #], Minjia Xie[1], Yuchen Tao[2], Ziqian Yin[1], Menghan Wang[1], Chao Ma[1], Zhouqing Chen[1,*], Zhong Wang[1, *]

[1]Department of Neurology, The First Affiliated Hospital of Soochow University, Suzhou 215006, Jiangsu Province, China

[2]Suzhou Medical College of Soochow University, Suzhou, Jiangsu Province, 215002, China

*** Correspondence:**

Zhong Wang

Email address: wangzhong761@163.com

Zhouqing Chen

Email address: zqchen6@163.com

Supplemental Figures

Supplementary Figure S1. Scatter plot of MR results. (A) scater plot of genetic correlations of 9 GM taxa and CAE using different MR methods; (B) scatter plot of genetic correlations of 10 GM taxa and JAE using different MR methods; (C) scatter plot of genetic correlations of 5 GM taxa and FE using different MR methods; (D) scatter plot of genetic correlations of 6 GM taxa and GE using different MR methods.

Supplementary Figure S2. Leave-one-out analysis for subtypes of epilepsy(A) 9 GM taxa on CAE, (B) 10 GM taxa on GAE, (C) 5 GM taxa on FE and (D) 6 GM taxa on GE

Supplementary Figure S3: PPI network of significant GM's SNPs mapped genes. Genes are represented by circular nodes, and their interactions are represented by edges. The size of the dots represents the Betweenness Centrality score, with high Betweenness Centrality scores being large and low scoring dots being small, and each of the bacteria related genes is represented by different colors. (A) PPI network of five significant GM's SNPs mapped genes of FE. (B) PPI network of six significant GM's SNPs mapped genes of GE. (C) PPI network of nine significant GM's SNPs mapped genes of CAE. (D) PPI network of ten significant GM's SNPs mapped genes of JAE.

Supplementary Figure S1. Scatter plot of MR results. (A) scater plot of genetic correlations of 9 GM taxa and CAE using different MR methods. (B) scatter plot of genetic correlations of 10 GM taxa and JAE using different MR methods.(C) scatter plot of genetic correlations of 5 GM taxa and FE using different MR methods.(D) scatter plot of genetic correlations of 6 GM taxa and GE using different MR methods

A. CAE

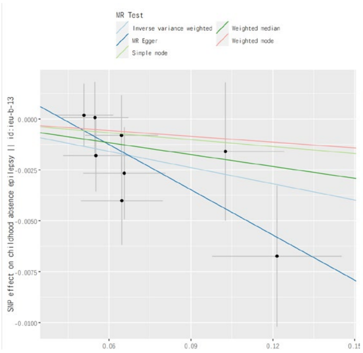

SNP effect on || id: class Bacteroidia id.912

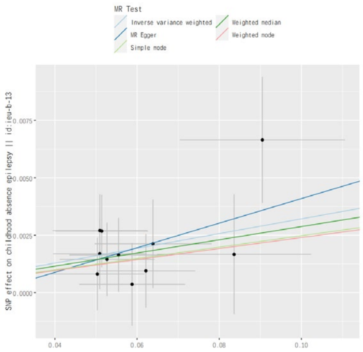

SNP effect on || id: family Veillonellaceae id.2172

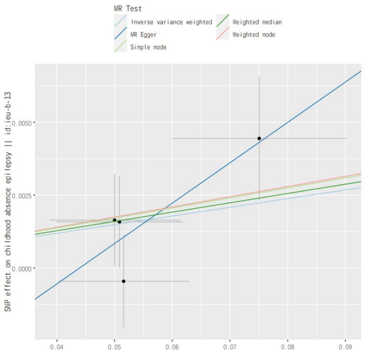

SNP effect on || id: genus Anaerostipes id.1991

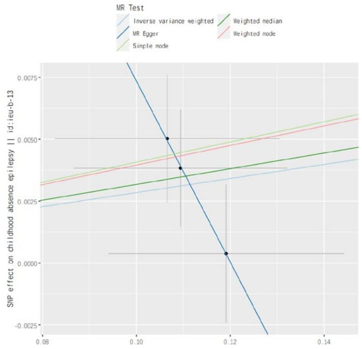

SNP effect on || id: genus Desulfovibrio id.3173

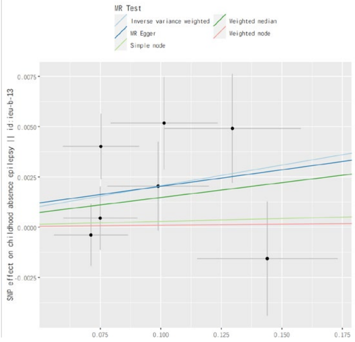

SNP effect on || id: genus Oscillibacter id.2063

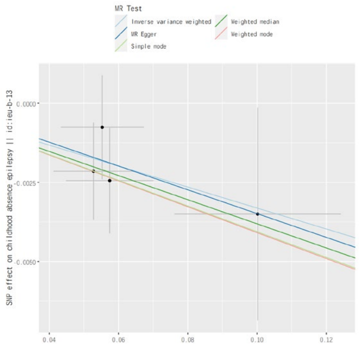

SNP effect on || id: genus Ruminococcaceae NK4A214 group id.11358

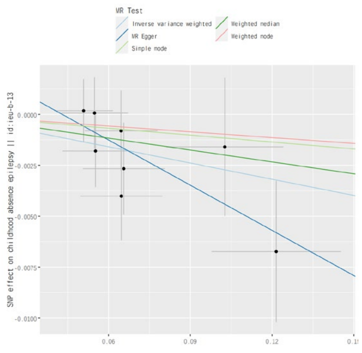

SNP effect on || id: order Bacteroidales id.913

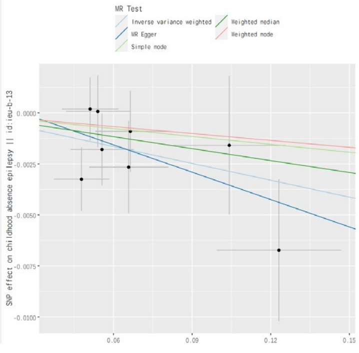

SNP effect on || id: phylum Bacteroidetes id.905

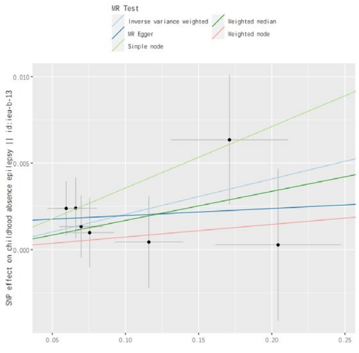

SNP effect on || id: phylum Verrucomicrobia id.3982

B. JAE

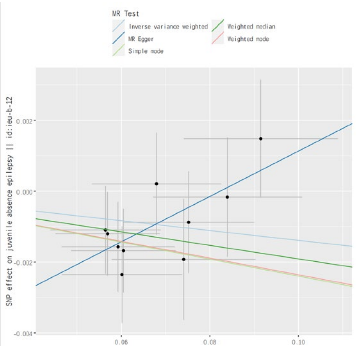

SNP effect on || id: family Prevotellaceae id.960

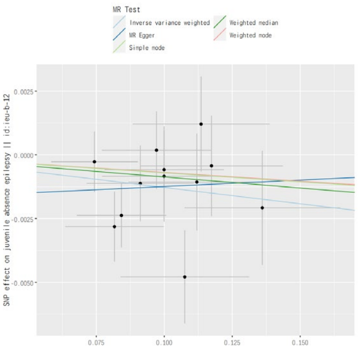

SNP effect on || id: family Rhodospirillaceae id.2717

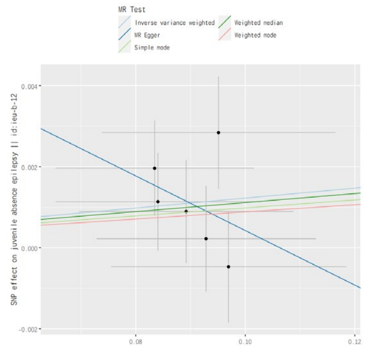

SNP effect on || id: genus Candidatus Soleaferrea id.11350

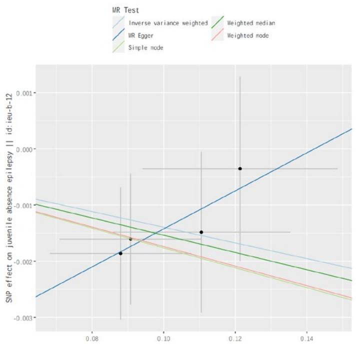

SNP effect on || id: genus Eggerthella id.819

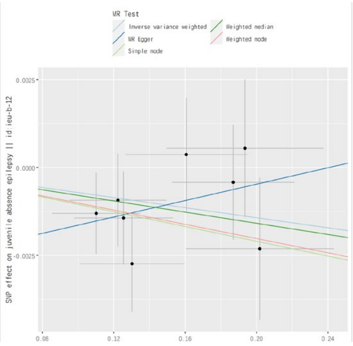

SNP effect on || id: genus Eubacterium nodatum group id.11297

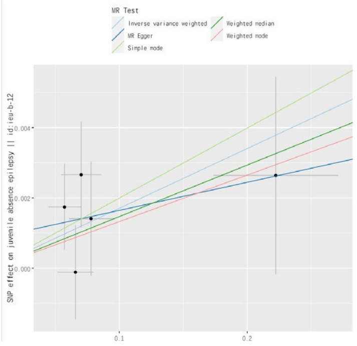

SNP effect on || id: genus Lachnospiraceae UCG010 id.11330

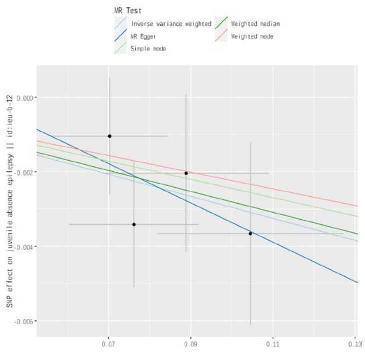

SNP effect on || id: genus Parabacteroides id.954

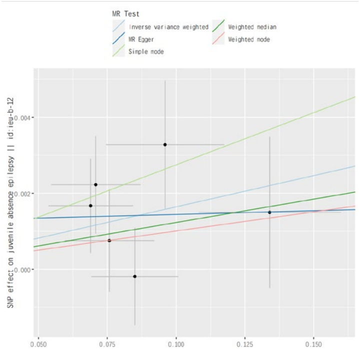

SNP effect on || id: genus Ruminococcaceae UCG004 id.11362

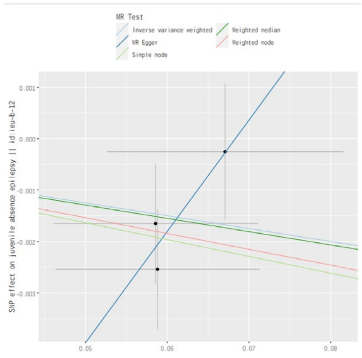

SNP effect on || id: genus Ruminococcaceae UCG010 id.11367

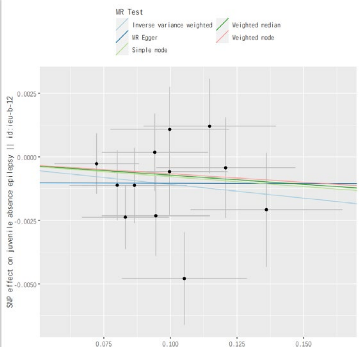

SNP effect on || id: order Rhodospirillales id.2667

C. FE

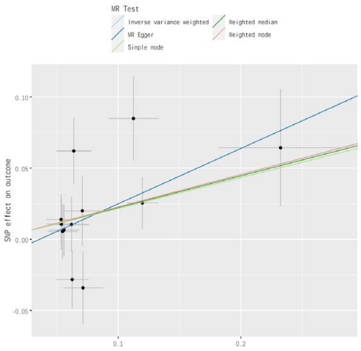

SNP effect on || id: genus Bifidobacterium id.436

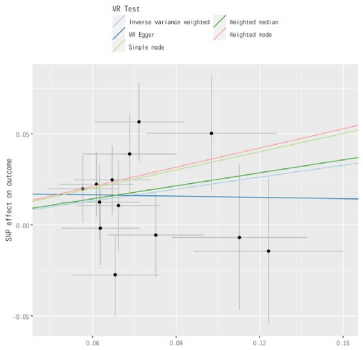

SNP effect on || id: genus Bilophila id.3170

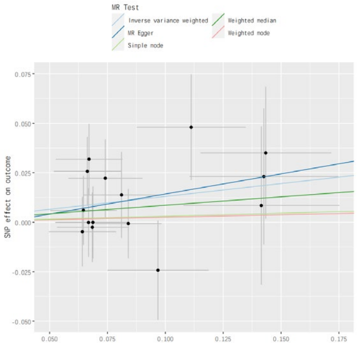

SNP effect on || id: genus Erysipelatoclostridium id.11381

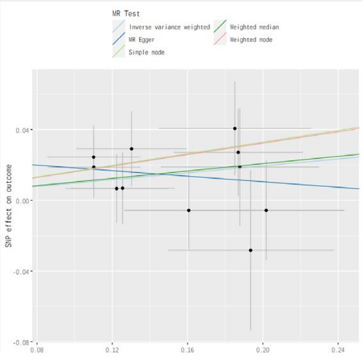

SNP effect on || id: genus Eubacterium nodatum group id.11297

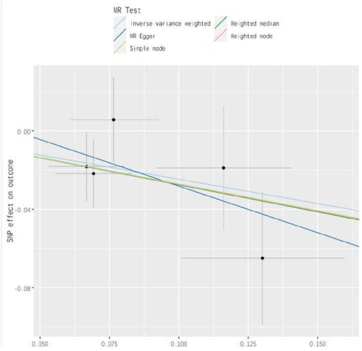

SNP effect on || id: genus Flavonifractor id.2059

D. GE

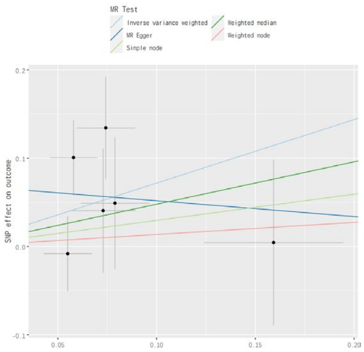

SNP effect on || id: class Gammaproteobacteria id.3303

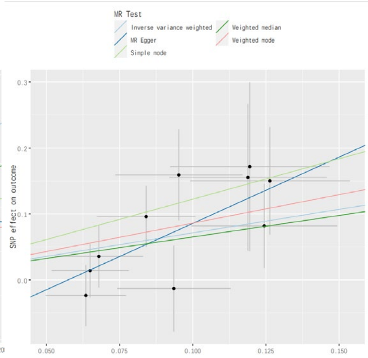

SNP effect on || id: genus Oxalobacter id.2978

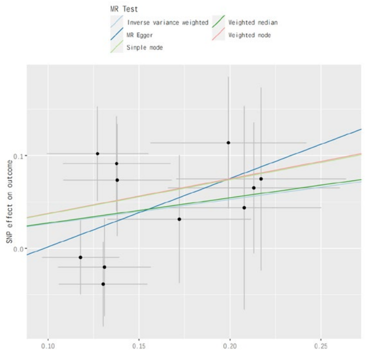

SNP effect on || id: genus Ruminococcaceae UCG013 id.11370

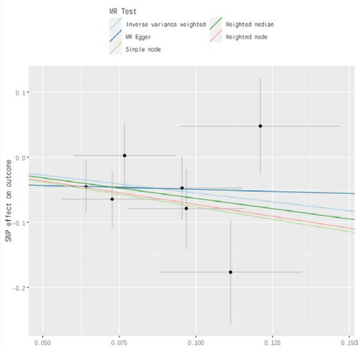

SNP effect on || id: genus Phascolarctobacterium id.2168

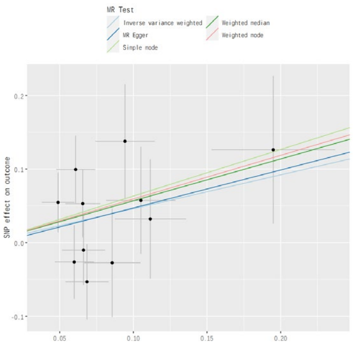

SNP effect on || id: genus Marvinbryantia id.2005

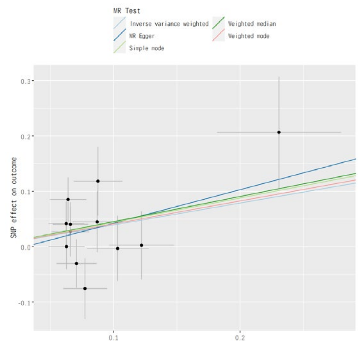

SNP effect on || id: order Mollicutes RF9 id.11579

Supplementary Figure S2. Leave-one-out analysis for (A) 9 GM taxa on CAE, (B) 10 GM taxa on GAE, (C) 5 GM taxa on FE and (D) 6 GM taxa on GE

A. CAE

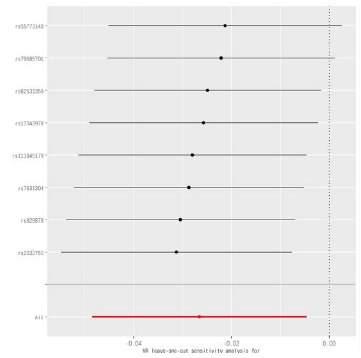

'|| id: class Bacteroidia id.912' on 'childhood absence epilepsy || id:ieu-b-13'

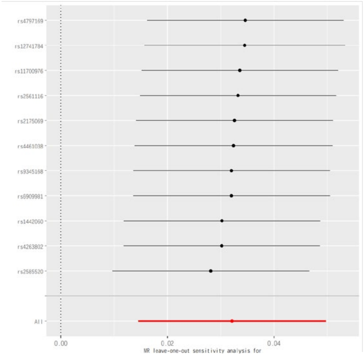

'|| id: family Veillonellaceae id.2172 ' on 'childhood absence epilepsy || id:ieu-b-13'

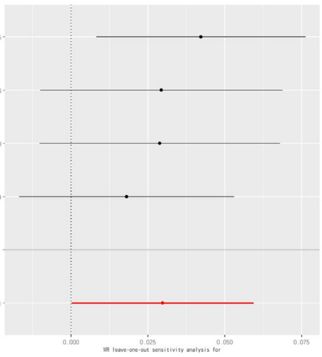

'|| id: genus Anaerostipes id.1991 ' on 'childhood absence epilepsy || id:ieu-b-13'

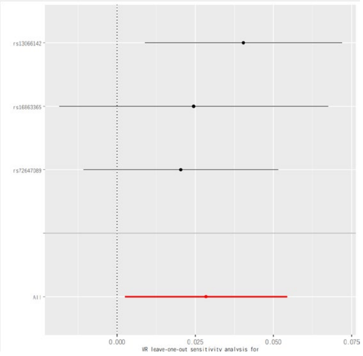

'|| id: genus Desulfovibrio id.3173' on 'childhood absence epilepsy || id:ieu-b-13'

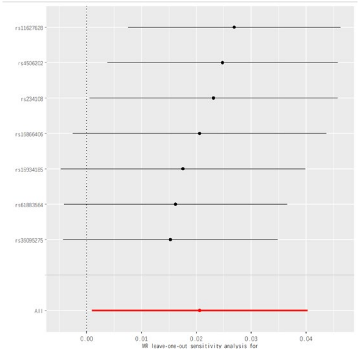

'|| id: genus Oscillibacter id.2063 ' on 'childhood absence epilepsy || id:ieu-b-13'

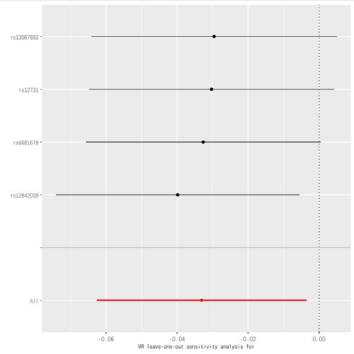

'|| id: genus Ruminococcaceae NK4A214 group id.11358 ' on 'childhood absence epilepsy || id:ieu-b-13'

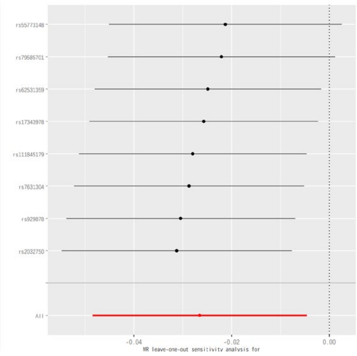

'|| id: order Bacteroidales id.913' on 'childhood absence epilepsy || id:ieu-b-13'

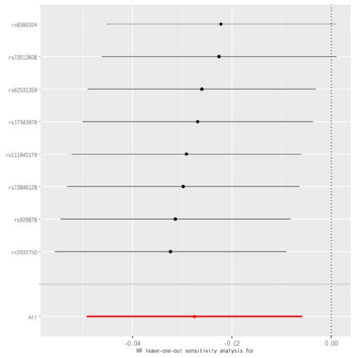

'|| id: phylum Bacteroidetes id.905 ' on 'childhood absence epilepsy || id:ieu-b-13'

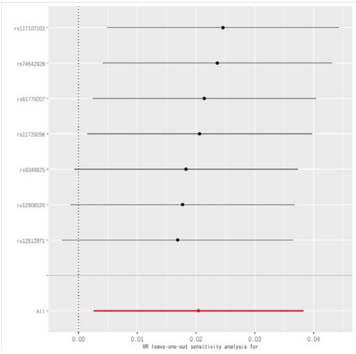

'|| id: phylum Verrucomicrobia id.3982 ' on 'childhood absence epilepsy || id:ieu-b-13'

B. JAE

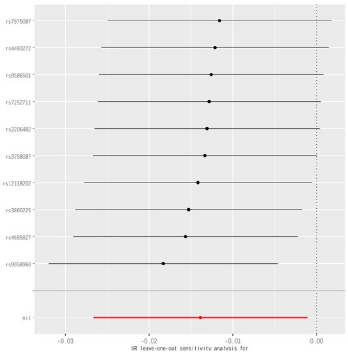

'|| id: family Prevotellaceae id.960' on 'childhood absence epilepsy || id:ieu-b-13' b-13'

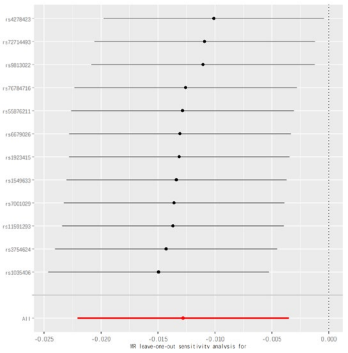

'|| id: family Rhodospirillaceae id.2717 ' on 'childhood absence epilepsy || id:ieu-b-13'

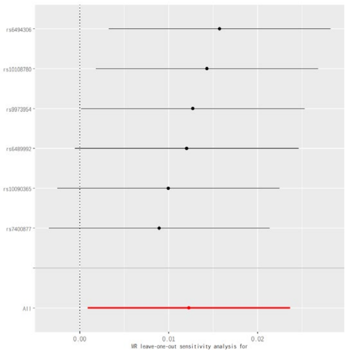

'|| id: genus Candidatus Soleaferrea id.11350 ' on 'childhood absence epilepsy || id:ieu-

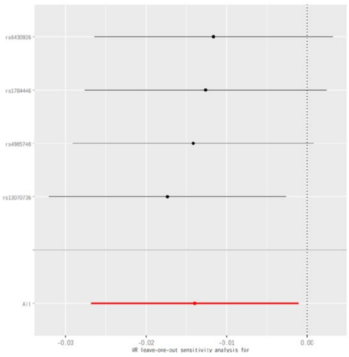

'|| id: genus Eggerthella id.819' on 'childhood absence epilepsy || id:ieu-b-13' id:ieu-b-13'

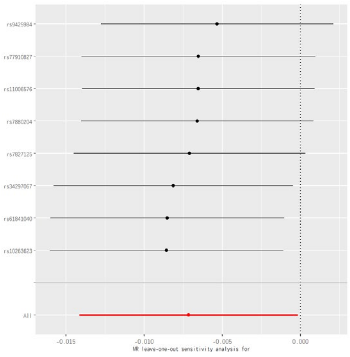

'|| id: genus Eubacterium nodatum group id.11297 ' on 'childhood absence epilepsy || id:ieu-b-13'

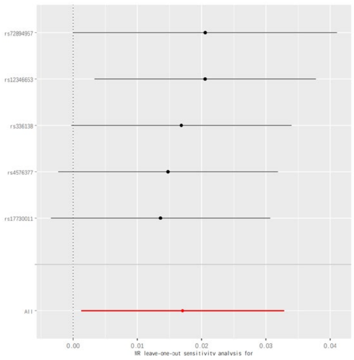

'|| id: genus Lachnospiraceae UCG010 id.11330 ' on 'childhood absence epilepsy ||

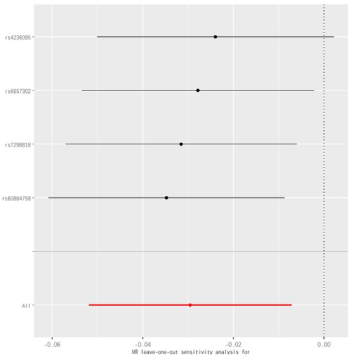

'|| id: genus Parabacteroides id.954' on 'childhood absence epilepsy || id:ieu-b-13' 13'

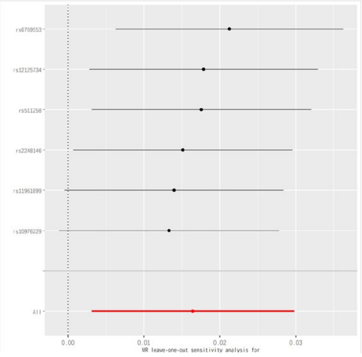

'|| id: genus Ruminococcaceae UCG004 id.11362 ' on 'childhood absence epilepsy || id:ieu-b-13'

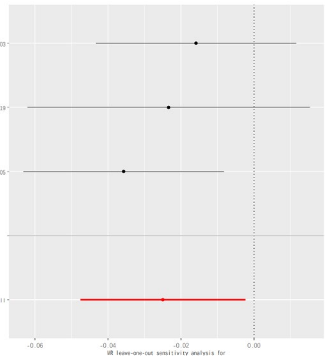

'|| id: genus Ruminococcaceae UCG010 id.11367 ' on 'childhood absence epilepsy || id:ieu-b-

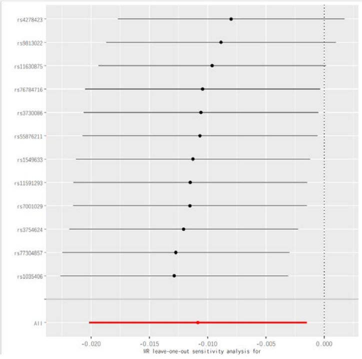

'|| id: order Rhodospirillales id.2667' on 'childhood absence epilepsy || id:ieu-b-13'

C. FE

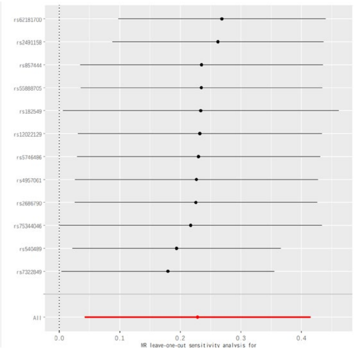

'|| id: genus Bifidobacterium id.436' on 'childhood absence epilepsy || id:ieu-b-13'

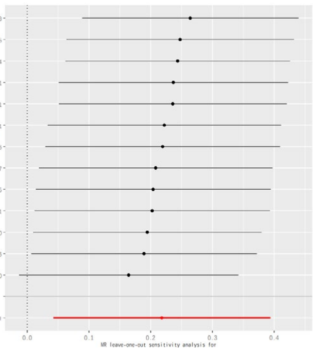

'|| id: genus Bilophila id.3170 ' on 'childhood absence epilepsy || id:ieu-b-13'

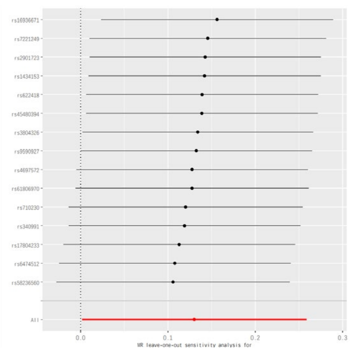

'|| id: genus Erysipelatoclostridium id.11381 ' on 'childhood absence epilepsy || id:ieu-b-13'

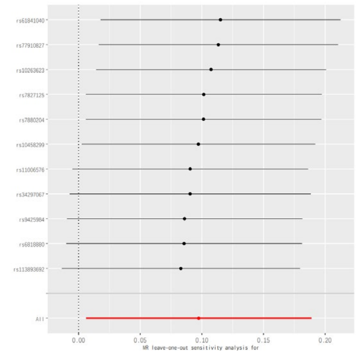

'|| id: genus Eubacterium nodatum group id.11297' on 'childhood absence epilepsy || id:ieu-b-13'

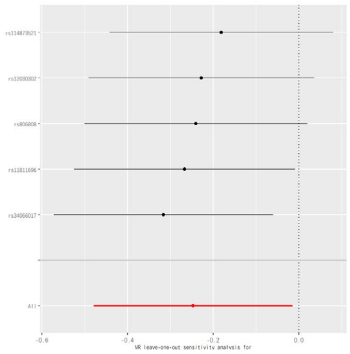

'|| id: genus Flavonifractor id.2059' on 'childhood absence epilepsy || id:ieu-b-13'

D. GE

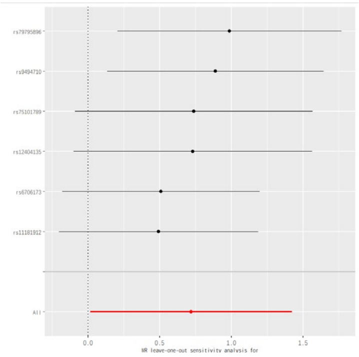

'|| id: class Gammaproteobacteria id.3303' on 'childhood absence epilepsy || id:ieu-b-13'

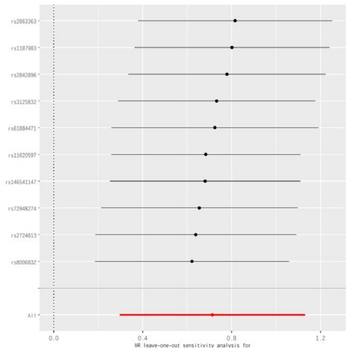

'|| id: genus Oxalobacter id.2978 ' on 'childhood absence epilepsy || id:ieu-b-13'

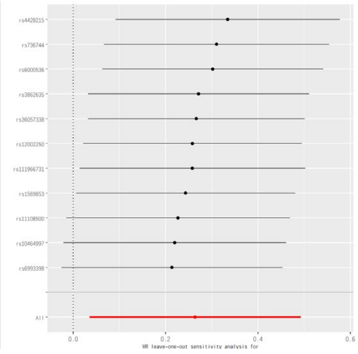

'|| id: genus Ruminococcaceae UCG013 id.11370 ' on 'childhood absence epilepsy || id:ieu-b-13'

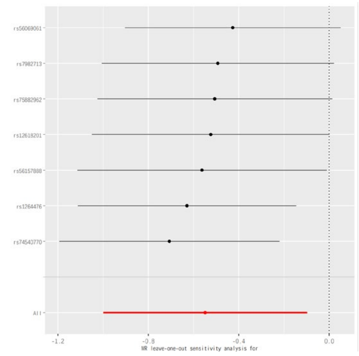

'|| id: genus Phascolarctobacterium id.2168' on 'childhood absence epilepsy || id:ieu-b-13'

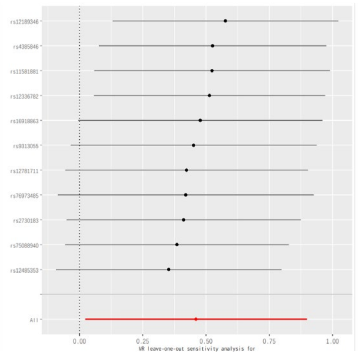

'|| id: genus Marvinbryantia id.2005 ' on 'childhood absence epilepsy || id:ieu-b-13'

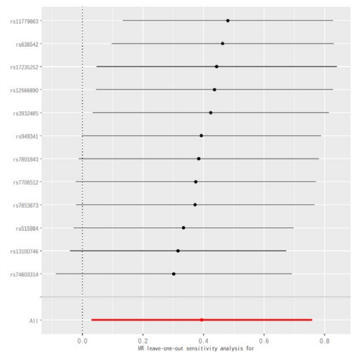

'|| id: order Mollicutes RF9 id.11579' on 'childhood absence epilepsy || id:ieu-b-13'

Supplementary Figure S3: PPI network of significant GM's SNPs mapped genes. Genes are represented by circular nodes, and their interactions are represented by edges. The size of the dots represents the Betweenness Centrality score, with high Betweenness Centrality scores being large and low scoring dots being small, and each of the bacteria related genes is represented by different colors. (A) PPI network of five significant GM's SNPs mapped genes of FE. (B) PPI network of six significant GM's SNPs mapped genes of GE. (C) PPI network of nine significant GM's SNPs mapped genes of CAE. (D) PPI network of ten significant GM's SNPs mapped genes of JAE.

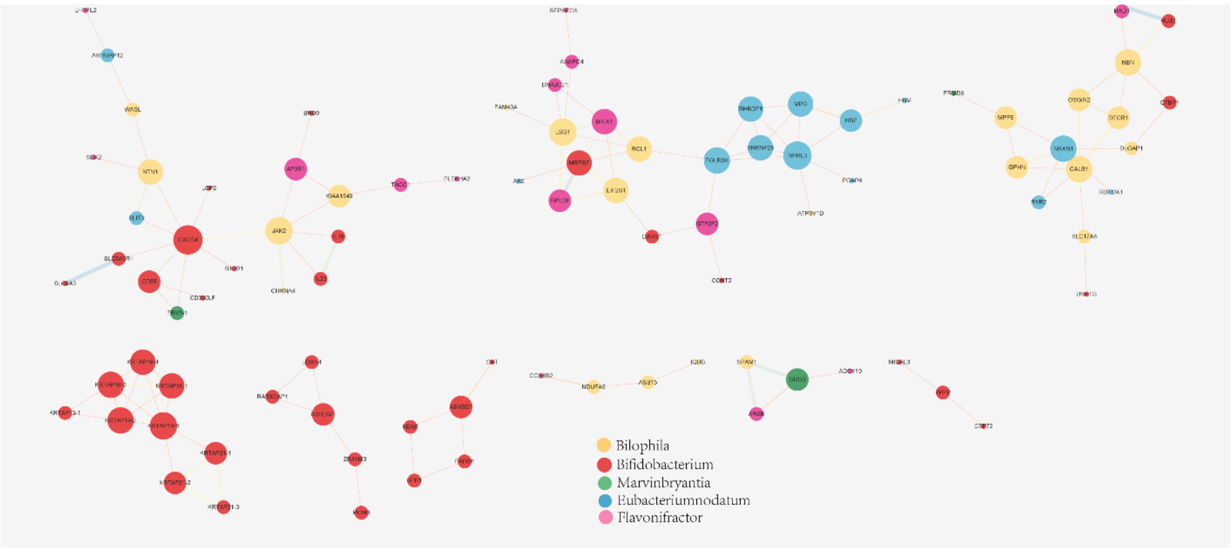

(A) PPI network of five significant GM's SNPs mapped genes of FE.

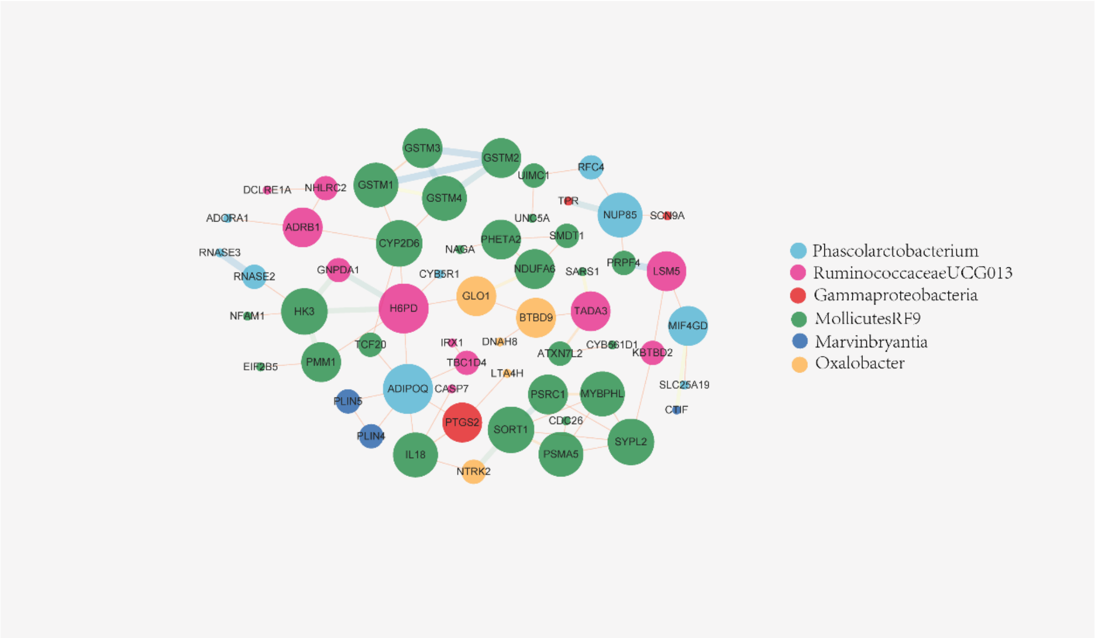

(B) PPI network of six significant GM's SNPs mapped genes of GE.

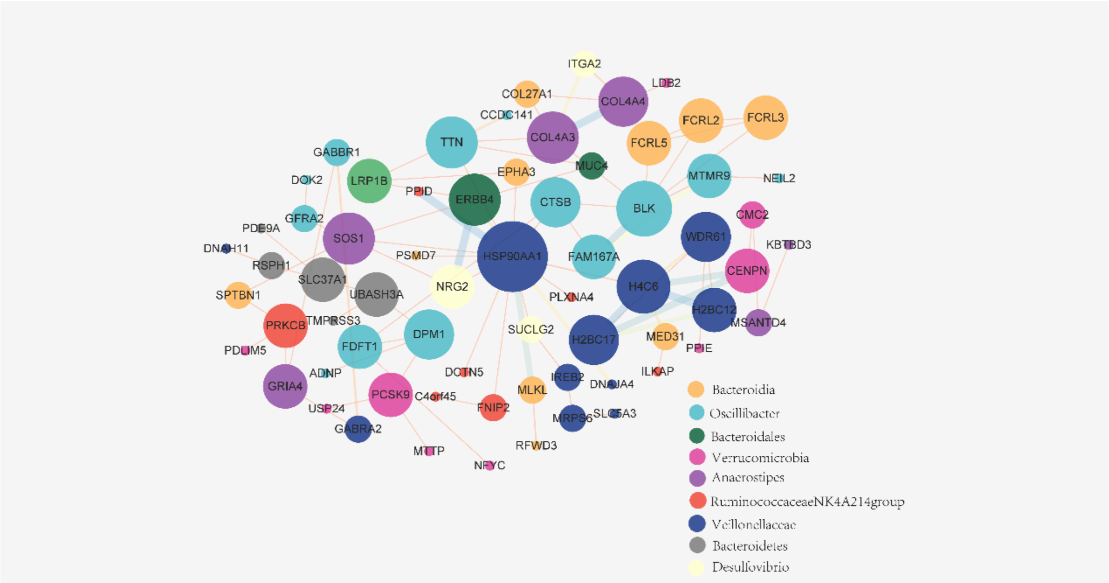

(C) PPI network of nine significant GM's SNPs mapped genes of CAE.

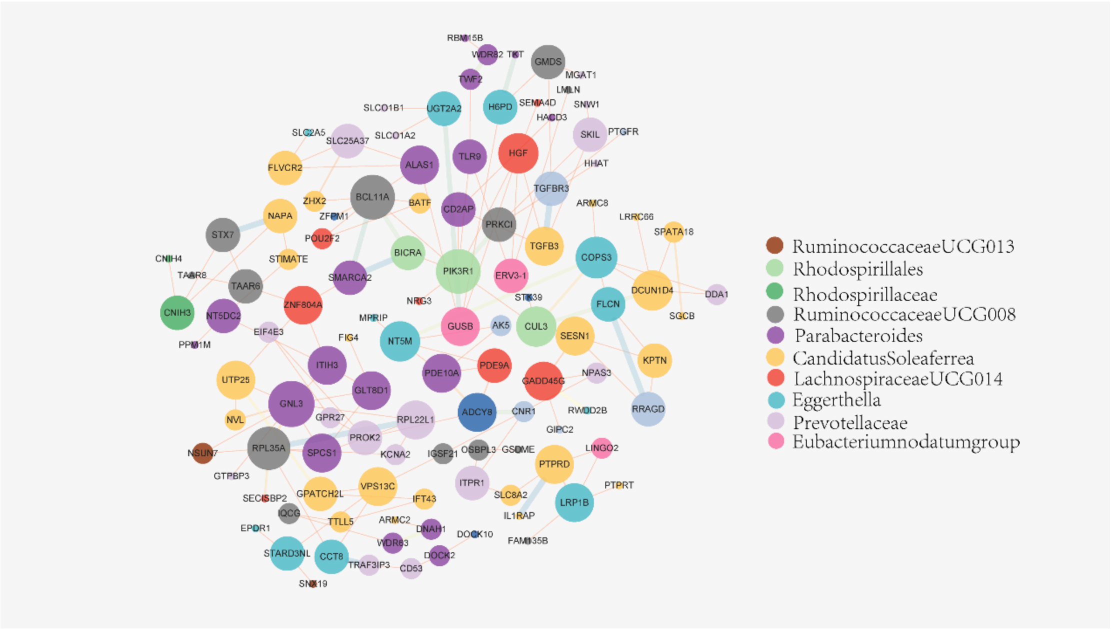

(D) PPI network of ten significant GM's SNPs mapped genes of JAE.
